# Salivary cardiac troponin does not correlate with serum levels

**DOI:** 10.3389/fcvm.2024.1440138

**Published:** 2025-01-07

**Authors:** Hoa Tran, Vu Hoang Vu, Quang Dang Duy Pham, Duc Minh Tran, Suong Thi Bang Nguyen, Vien Thanh Truong, Binh Quang Truong

**Affiliations:** ^1^ Faculty of Medicine, University of Medicine and Pharmacy at Ho Chi Minh City, Ho Chi Minh City, Vietnam; ^2^ Interventional Cardiology Department, University Medical Center Ho Chi Minh City, Ho Chi Minh City, Vietnam; ^3^ Laboratory Department, University Medical Center Ho Chi Minh City, Ho Chi Minh City, Vietnam; ^4^ Department of Cardiology, The Christ Hospital Health Network, Lindner Research Center, Cincinnati, OH, United States

**Keywords:** salivary troponin, serum troponin, correlation, myocardial injury, chest discomfort

## Abstract

**Introduction:**

Several studies suggest a potential correlation between troponin levels detected in serum and saliva. However, prior investigations have not adequately addressed the critical aspect of collecting samples upon admission, which is essential for timely troponin level determination. This study aimed to evaluate the relationship between troponin levels in serum and saliva among patients admitted for chest pain evaluation.

**Methods:**

This observational study was conducted at the Interventional Cardiology Department of the University Medical Center HCMC. Patients presenting with chest pain were enrolled, and unstimulated saliva samples were collected using the Navazesh method simultaneously with the initial blood collection. These samples were then analyzed for levels of salivary troponin I, serum troponin I, and serum high-sensitive troponin T.

**Results:**

Among the 48 patients included, 22 (46%) exhibited myocardial injury, while 12 (25%) were diagnosed with acute myocardial infarction. No significant difference was observed in salivary troponin I levels between the non-myocardial injury and myocardial injury groups (*p* = 0.425). Moreover, no correlation was found between salivary troponin I levels and either serum troponin T or serum troponin I levels (Pearson correlation *p* = 0.761, 0.500; Spearman correlation *p* = 0.857, 0.136, respectively). The ROC curve for salivary troponin I in predicting myocardial injury displayed an AUC of 0.566 (95% CI: 0.402–0.731), indicating poor discriminatory power.

**Conclusions:**

In our investigation, salivary troponin I failed to demonstrate a meaningful correlation with serum troponins, thereby limiting its practical utility in diagnosing myocardial injury or myocardial infarction. Further research is warranted to explore its diagnostic reliability and clinical applicability.

## 1 Introduction

Serum troponin has become a cornerstone in the diagnosis of acute myocardial infarction due to its high sensitivity and specificity (1). Moreover, serum troponin levels have proven to be valuable prognostic indicators, aiding in risk stratification, and predicting adverse cardiac events in patients with acute myocardial infarction (2). Recent studies have hinted at the existence of a correlation between troponin levels detected in serum and saliva (3–5). If substantiated, these findings hold promise for offering a more convenient and timely approach in assisting with the diagnosis and prognosis of patients diagnosed with acute coronary syndrome. However, previous studies have not accounted for the collection of saliva and serum samples upon admission, which is crucial in real-world scenarios where prompt determination of troponin levels is essential. We conducted a study aiming to assess the relationship between troponin levels in both serum and saliva among patients admitted to the cardiology department for chest pain evaluation, and to explore the potential of salivary troponin in diagnosing myocardial injury and/or myocardial infarction.

## 2 Methods

### 2.1 Study design and participants

In this observational study, patients admitted to the Interventional Cardiology Department at the University Medical Center Ho Chi Minh City from October 2021 to August 2022, to evaluate chest pain, met the inclusion and exclusion criteria described below and were invited to participate. After informed consent was obtained, patients were instructed to provide unstimulated saliva samples using the Navazesh method, ideally within 10 min of the first prescribed blood collection. The collected samples were then delivered to the central biochemical lab for analysis of salivary troponin I, serum troponin I, and serum high-sensitive troponin T concentrations, among other serum tests ordered by the attending physician. Additional relevant information, including age, previous medical history, complete blood count, serial serum high-sensitive troponin T, creatine kinase-MB (CK-MB), urea, creatinine, aspartate aminotransferase (AST), alanine aminotransferase (ALT), C-reactive protein (CRP), and N-terminal pro–B-type natriuretic peptide (NT-proBNP) levels, as well as the final discharge diagnosis, was gathered from the patients' electronic medical records.

According to the Fourth Universal Definition of Myocardial Infarction, detection of an elevated cTn value above the 99th percentile upper reference limit is defined as myocardial injury (1). In our study, myocardial injury is determined by elevated high-sensitive troponin T to the level of over 14 ng/dl. Patients with myocardial injury may or may not have myocardial infarction. Myocardial infarction is defined as detection of a rise and/or fall of cardiac troponin values with at least 1 value above the 99th percentile upper reference limit and with at least 1 of the following: symptoms of acute myocardial ischemia; new ischemic ECG changes; development of pathological Q waves; imaging evidence of new loss of viable myocardium or new regional wall motion abnormality in a pattern consistent with an ischemic etiology; identification of a coronary thrombus by angiography including intracoronary imaging or by autopsy (1).

Quantitative measurement of high-sensitive serum troponin T was conducted at the Laboratory Department of the University of Medicine and Pharmacy Hospital using the Roche Elecsys hs Troponin T assay on the Roche Cobas e801 analyzer. Quantitative measurement of serum troponin I and salivary troponin I was performed using an ELISA method based on the Cloud-Clone Corp kit (Katy, TX 77494, USA; https://www.cloud-clone.us) at the same Laboratory Department.

#### 2.1.1 Inclusion, exclusion criteria

Inclusion criteria: Male or female of at least 18 years of age, had chest discomfort as chief complaint.

Exclusion criteria:

1. Patients had acute respiratory failure, shock, active infection.
2. Patients had oral diseases
3. Patients had eaten or drink within 30 min before blood sample taking.
4. Patients cannot take salivary sample as instructed.

#### 2.1.2 Saliva sample collecting procedure

The patient's saliva samples were collected non-stimulated using the Navazesh method (6), simultaneously with the collection of the prescribed blood samples:

• The nurse provides the patient with a large vial for collecting saliva, which is labeled with the patient's identification code. This vial is referred to as the “saliva collection vial”.
• The patient rinses their mouth with filtered water and spits out or swallows any saliva.
• Within the next 5 min, the patient spits all the saliva from their mouth into this “saliva collection vial”. No eating or drinking is allowed during this 5 min period. The vial is closed tightly after collection.
• The patient can resume normal eating and drinking habits after collecting the saliva (e.g., drinking milk, taking medication, etc.).

### 2.2 Sample size and sampling

The sample size for this study was determined to evaluate the correlation between salivary troponin and serum troponin levels using Pearson's correlation coefficient. We aimed for a statistical power of 80% and a significance level of 0.05. Using the formula for sample size calculation for correlation analysis by Machin D et al. (7), we obtained a minimum required sample size of 29 pairs of observations. Convenience sampling was utilized as the sampling method in this study.

### 2.3 Statistical method

Data was analysed using R version 4.3.3. Data were presented as mean ± SD, median (interquartile range), or number (%), as appropriate. Baseline characteristics were compared between subjects with and without myocardial injury using *χ*^2^ tests for categorical variables and 2-sided t tests or the Wilcoxon signed-rank test for continuous variables. Variables with non-normal distribution were transformed to logarithmic values. Pearson and Spearman correlation coefficients were used to assess relationships between continuous variables. Receiver operating characteristic (ROC) curve analysis was conducted to assess the diagnostic performance of salivary troponin I levels in predicting myocardial injury and myocardial infarction. The best cut-off points for salivary troponin I levels were chosen using the Youden Index, which maximizes the sum of sensitivity and specificity. All statistical tests were two-tailed, and *p*-values less than 0.05 were considered statistically significant.

### 2.4 Ethical considerations

The study protocol was reviewed and approved by the Institutional Review Board (IRB)/Ethics Committee of University Medical Center HCMC, with the approval number 57/GCN-HDDD. All participants provided written informed consent prior to their enrollment in the study.

## 3 Results

**Figure 1:**
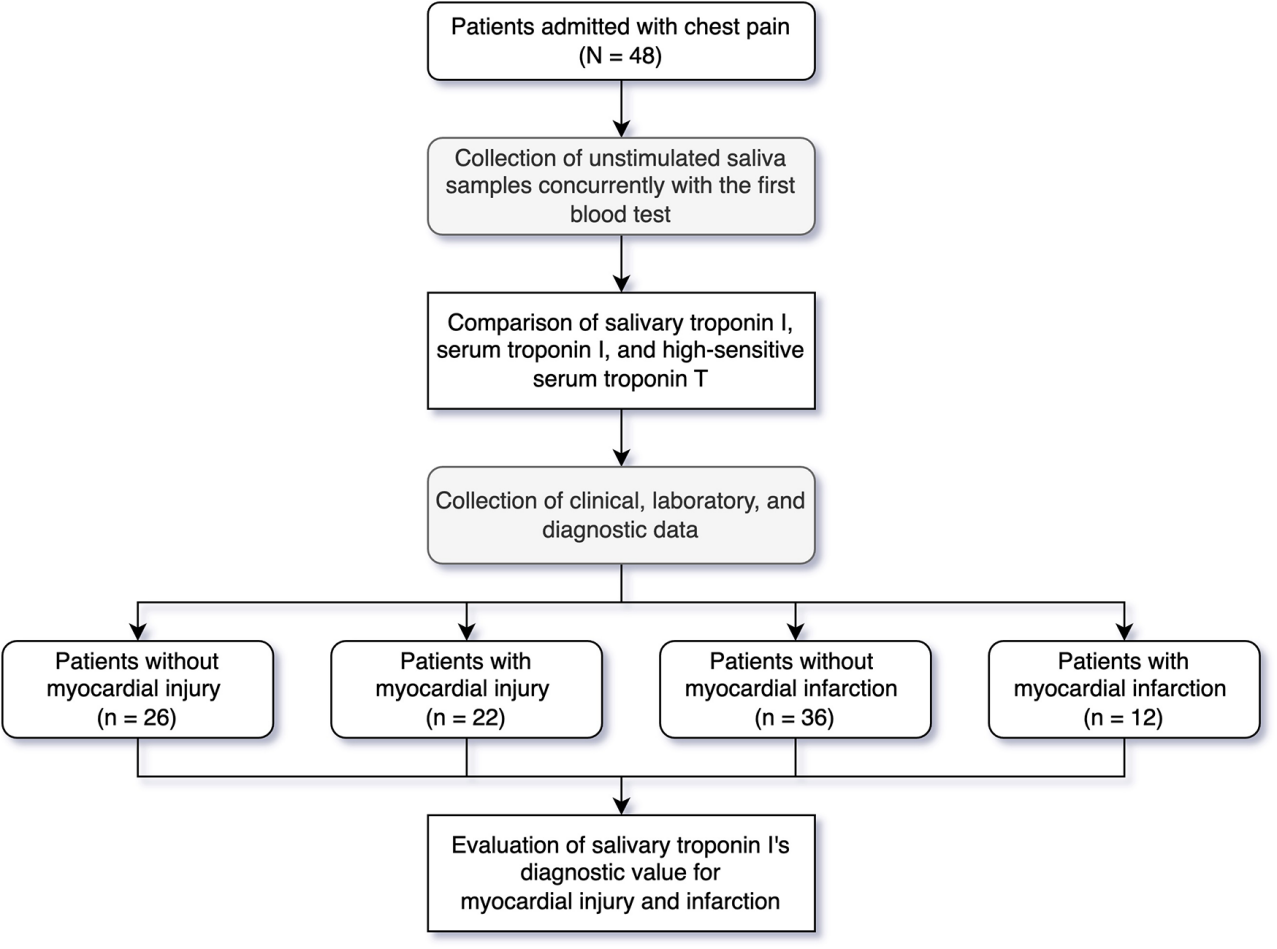
Study profile.

Figure 1 illustrates the study profile. A total of 48 patients were enrolled, with 22 (46%) showing evidence of myocardial injury. Table 1 summarizes the patient characteristics. The mean age was 61.0 ± 10.0 years, and 31% of the cohort were female. The median time from symptom onset to saliva collection was 5 days (IQR: 3.5–7). The myocardial injury group had a significantly higher prevalence of diabetes and acute kidney injury (*p* = 0.006 and 0.030, respectively), as well as elevated serum AST and NT-proBNP levels (both *p* < 0.001). There were no significant differences between the two groups in terms of symptom onset time, CK-MB, white blood cell count (WBC), hemoglobin (HGB), platelet count (PLT), serum urea, creatinine, ALT, or NT-proBNP. Within the myocardial injury group, 12 (55%) patients were diagnosed with acute myocardial infarction. Notably, there was no significant difference in salivary troponin I levels between the non-myocardial injury and myocardial injury groups (*p* = 0.425). Correlation analyses (Pearson and Spearman) were conducted to assess the relationship between symptom onset time and salivary troponin I levels, revealing no significant correlation (Pearson *p* = 0.328, Spearman *ρ* = 0.839).

**Table 1:** Patients characteristics.

	Total (*N* = 48)	Non-myocardial injury (*n* = 26)	Myocardial injury (*n* = 22)	*p* value
Age	61.0 ± 10.0	61.1 ± 8.0	63.1 ± 12.9	0.078
Gender, female	31%	39%	23%	0.241
Symptoms onset (days)	5.0 (3.5–7)	4.0 (3.4–6.0)	6.0 (4.0–7.0)	0.109
Hypertension	79%	81%	77%	0.564
Diabetes	19%	12%	27%	**0**.**006**
Current smoker	15%	12%	18%	0.413
Acute kidney injury	2%	0%	5%	**0**.**030**
CK-MB (U/L)	15 (12–21)	14 (10–17)	16 (13–23)	0.217
Serum high-sensitive troponin T (pg/ml)	11.5 (8.2–42.0)	8.3 (7.0–10.2)	47.2 (26.1–534.0)	**<0**.**001**
Serum troponin I (pg/ml)	10.0 (10.0–28.1)	10 (10–10)	27.9 (13.2–392.1)	**<0**.**001**
Salivary troponin I (pg/ml)	22.9 (0.0–54.7)	36.6 (0.0–62.2)	17.0 (0.0–35.0)	0.425
WBC (G/L)	7.6 ± 1.8	7.4 ± 1.6	7.8 ± 2.1	0.138
HGB (g/L)	136.2 ± 13.4	137.7 ± 12.8	134.4 ± 14.1	0.507
PLT (G/L)	259.8 ± 66.1	253.5 ± 56.2	267.3 ± 76.9	0.250
Urea (mg/dl)	36.3 ± 18.1	35.5 ± 17.4	37.3 ± 19.4	0.703
Creatinin (mg/dl)	1.01 ± 0.32	0.95 ± 0.20	1.09 ± 0.41	0.218
AST (U/L)	40.4 ± 38.8	28.6 ± 11.1	54.6 ± 53.6	**<0**.**001**
ALT (U/L)	32.8 ± 21.1	31.4 ± 21.4	34.6 ± 21.2	0.899
NT-proBNP (ng/L)	303 (67–632)	73 (32–156)	669 (494–1,449)	**<0**.**001**
Acute myocardial infarction	12 (25%)	0 (0%)	12 (55%)	**<0**.**001**

*p* values less than 0.05 are considered statistically significant and are highlighted in bold.

**Figure 2:**
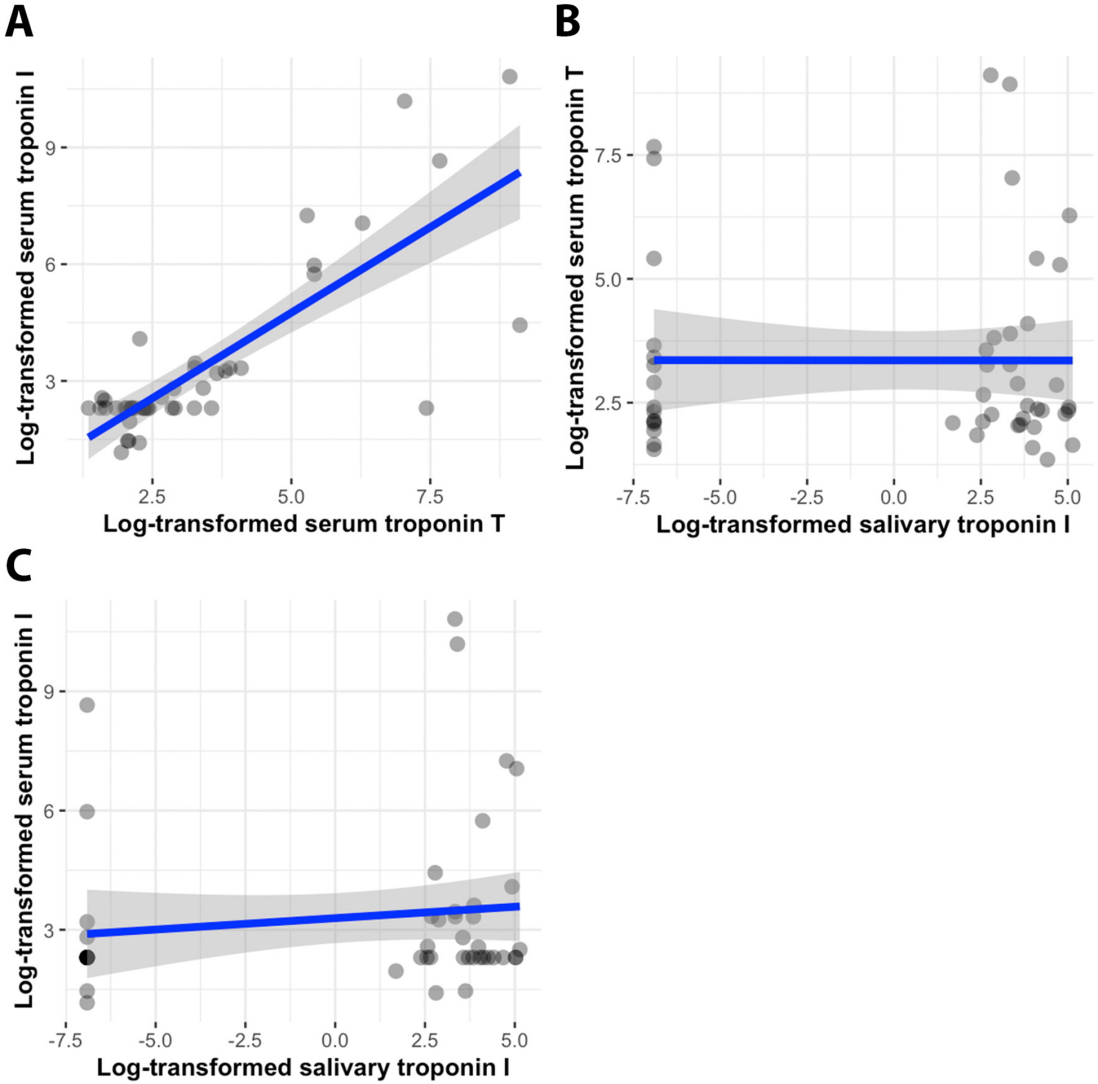
Scatterplots of serum troponin T–serum troponin I values **(A)**, salivary troponin I–serum troponin T values **(B)**, and salivary troponin I–serum troponin I values **(C)**, accompanied by regression lines (blue) and 95% confidence intervals of the standard deviation (grey). All values are log-transformed.

Pearson and Spearman correlation coefficients were calculated to assess the relationship between serum high-sensitive troponin T (serum TnT), serum troponin I (serum TnI), and unstimulated salivary troponin I (saliva TnI) (Table 2). As anticipated, a strong positive correlation was observed between serum troponin T and troponin I levels (Pearson correlation *r* = 0.584, *p* < 0.001; Spearman correlation *ρ* = 0.703, *p* < 0.001) (Figure 2A). However, there was no correlation found between salivary troponin I levels and either serum troponin T or serum troponin I levels (Pearson correlation *r* = −0.045, *p* = 0.761; *r* = −0.101, *p* = 0.500, respectively; Spearman correlation *ρ* = −0.027, *p* = 0.857; *ρ* = 0.218, *p* = 0.136, respectively) (Figures 2B,C). Partial correlation analysis further confirmed the absence of any correlation between salivary TnI levels and serum TnT or serum TnI levels in both the myocardial injury and non-myocardial injury groups (*p* = 0.679 for salivary TnI vs. serum TnT; *p* = 0.936 for salivary TnI vs. serum TnI).

**Table 2:** Correlations between serum high-sensitive troponin T, serum troponin I and unstimulated salivary troponin I.

	Pearson correlation r	*p* value	Spearman correlation *ρ*	*p* value
Serum troponin T–Serum troponin I	0.584	**<0**.**001**	0.703	**<0**.**001**
Salivary troponin I–Serum troponin T	−0.045	0.761	−0.027	0.857
Salivary troponin I–Serum troponin I	−0.101	0.500	0.218	0.136

*p* values less than 0.05 are considered statistically significant and are highlighted in bold.

**Figure 3:**
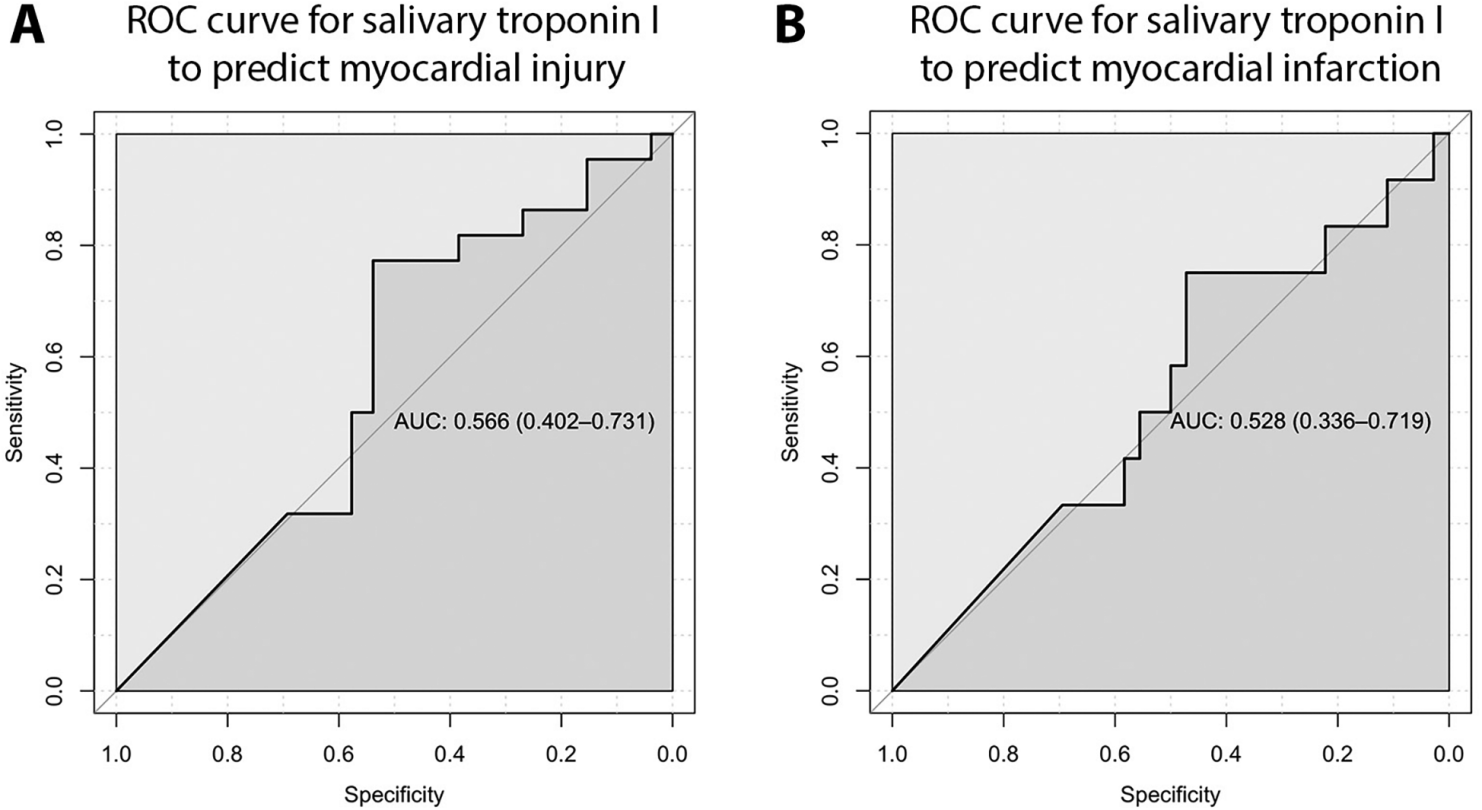
ROC curve for salivary troponin I to predict myocardial injury **(A)** and myocardial infarction **(B)**, demonstrating poor discriminatory power.

The ROC curve analysis for salivary troponin I in predicting myocardial injury yielded an AUC of 0.566 (95% CI: 0.402–0.731), demonstrating poor discriminatory power. Additionally, the ROC curve crossing the mid-line indicates that salivary troponin I is ineffective in differentiating between patients with and without myocardial injury (Figure 3A). Similarly, salivary troponin I does not effectively distinguish between patients with and without myocardial infarction, as evidenced by an AUC of 0.528 (95% CI: 0.336–0.719) (Figure 3B). The best salivary troponin I cut-off point for predicting myocardial injury is 35.25 pg/ml. At this cut-off point, the sensitivity and specificity of salivary troponin I to detect myocardial injury are 36.4% and 92.3%, respectively, with a positive predictive value of 80% and a negative predictive value of 63.2%. For predicting myocardial infarction, the optimal salivary troponin I cut-off point is 32.475 pg/ml, yielding a sensitivity and specificity of 66.7% and 94.4%, respectively, with a positive predictive value of 80% and a negative predictive value of 89.5%.

## 4 Discussion

The utilization of salivary troponin as an indicator for patients with myocardial infarction was initially introduced by Mirzaii-Dizgah et al. in two published papers. In the first study, the authors examined 30 patients with acute ST-elevation myocardial infarction and 28 normal healthy individuals. Serum and saliva samples were collected 12 and 24 h after presentation to the Emergency Department, and cardiac troponin I levels were assayed. The authors observed that both serum and unstimulated saliva concentrations of troponin I were significantly elevated in patients with acute ST-elevation myocardial infarction compared to controls (3). In the second study, the authors conducted an examination involving 30 patients diagnosed with acute ST-elevation myocardial infarction, along with 30 normal healthy individuals. High-sensitive troponin T levels were assessed in both serum and saliva samples collected during the initial and subsequent mornings following hospital admission. The results unveiled a notable elevation in the mean levels of both stimulated and unstimulated saliva, as well as serum levels of high-sensitive troponin T, among patients with acute ST-elevation myocardial infarction in comparison to the healthy control group (4). While serum troponin levels are significantly elevated in the setting of acute ST-elevation myocardial infarction, and the utility of troponin testing in such scenarios remains debatable given the paramount importance of electrocardiography for timely diagnosis and management, nonetheless, these findings suggest a potential correlation between salivary and serum cardiac troponins.

Our study aimed to evaluate the effectiveness of salivary troponin as a diagnostic tool for managing patients presenting with chest discomfort, a context where serum troponin plays a pivotal role in diagnosis and prognosis. Our findings reveal a limited correlation between salivary troponin I and serum troponins. Specifically, salivary troponin I demonstrated weak correlations with both serum troponin I and T, limiting its diagnostic utility for myocardial injury. Importantly, this discrepancy cannot be attributed to inaccuracies in the troponin I kit test utilized, as serum troponin I levels determined by this kit exhibited a strong positive correlation with serum high-sensitive troponin T.

We hypothesize that salivary troponin levels may not rise until serum troponin levels are significantly elevated for a prolonged period, as observed in acute ST-elevation myocardial infarction settings, as reported by Mirzaii-Dizgah et al. This limitation affects the practical applicability of salivary troponin in diagnosing acute myocardial injury and/or myocardial infarction.

As a result, the diagnostic utility of salivary troponin I for myocardial injury and myocardial infarction is limited, as the test has no discriminative ability. In our study, the best salivary troponin I cut-off point for predicting myocardial injury is 35.25 pg/ml, which provides sensitivity and specificity of 36.4% and 92.3%, respectively. For predicting myocardial infarction, the optimal salivary troponin I cut-off point is 32.475 pg/ml, yielding sensitivity and specificity of 66.7% and 94.4%, respectively.

In a study by Miller CS et al., no significant difference was found between salivary and serum troponin I levels (*p* = 0.054) in patients with acute myocardial infarction (63% STEMI) and controls (8). In a recent study, R. Westreich et al. applied advanced saliva processing techniques, including Amplification Buffer, Hampering Remover, and a Saliva Concentrator Cartridge. Their results showed that processed salivary troponin I had a sensitivity of 84% and specificity of 100% for detecting myocardial injury, whereas unprocessed saliva demonstrated a much lower sensitivity of only 6.25% (5). These findings align with our study, where unprocessed, unstimulated saliva samples were collected.

This study has several limitations that should be acknowledged. First, the troponin I kit used was not of the “high-sensitivity” type. The timing of sample collection, performed only once at the time of admission, may not have captured potential changes in salivary troponin I levels over time or in response to acute myocardial events. Additionally, the study did not account for factors such as oral health status and hydration, which could influence the composition of salivary biomarkers. Despite these limitations, the study protocol was designed to mirror real-world clinical practice and underscored the challenges of using salivary troponin I as a diagnostic tool.

## 5 Conclusion

Salivary troponin may show a correlation with serum troponin, but this correlation may necessitate sophisticated salivary processing techniques or significantly prolonged elevated serum troponin levels. In our investigation, salivary troponin failed to demonstrate a meaningful correlation with serum troponin, thereby limiting its practical utility in real-world clinical settings. While the potential of salivary troponin remains promising, further research is warranted to explore its diagnostic reliability and clinical applicability.

## Data Availability

The raw data supporting the conclusions of this article will be made available by the authors, without undue reservation.
